# Correction and removal of expression of concern: Enhancement of anti-leukemic potential of 2-hydroxyphenyl-azo-2′-naphthol (HPAN) on MOLT-4 cells through conjugation with Cu(ii)

**DOI:** 10.1039/d4ra90149e

**Published:** 2024-12-12

**Authors:** Tathagata Deb, Priya Kalyan Gopal, Durba Ganguly, Piyal Das, Mausumi Paul, Manju Bikash Saha, Santanu Paul, Saurabh Das

**Affiliations:** a Department of Chemistry (Inorganic Section), Jadavpur University Kolkata 700 032 India dasrsv@yahoo.in +91 33 24146223 +91 8902087756; b Laboratory of Experimental Immunology, Department of Microbiology and Botany, Gurudas College Kolkata 700 054 India spaul_1971@yahoo.com +91 33 23546623 +91 9874192648; c Department of Chemistry, Indian Institute of Chemical Biology Kolkata 700 032 India

## Abstract

Correction and removal of expression of concern for ‘Enhancement of anti-leukemic potential of 2-hydroxyphenyl-azo-2′-naphthol (HPAN) on MOLT-4 cells through conjugation with Cu(ii)’ by Tathagata Deb *et al.*, *RSC Adv.*, 2014, **4**, 18419–18430, https://doi.org/10.1039/C3RA44765K.

The authors regret an error in the manuscript preparation where the data for Fig. 4b was incorrect. The original and correct Fig. 4b is shown below.

**Fig. 1:**
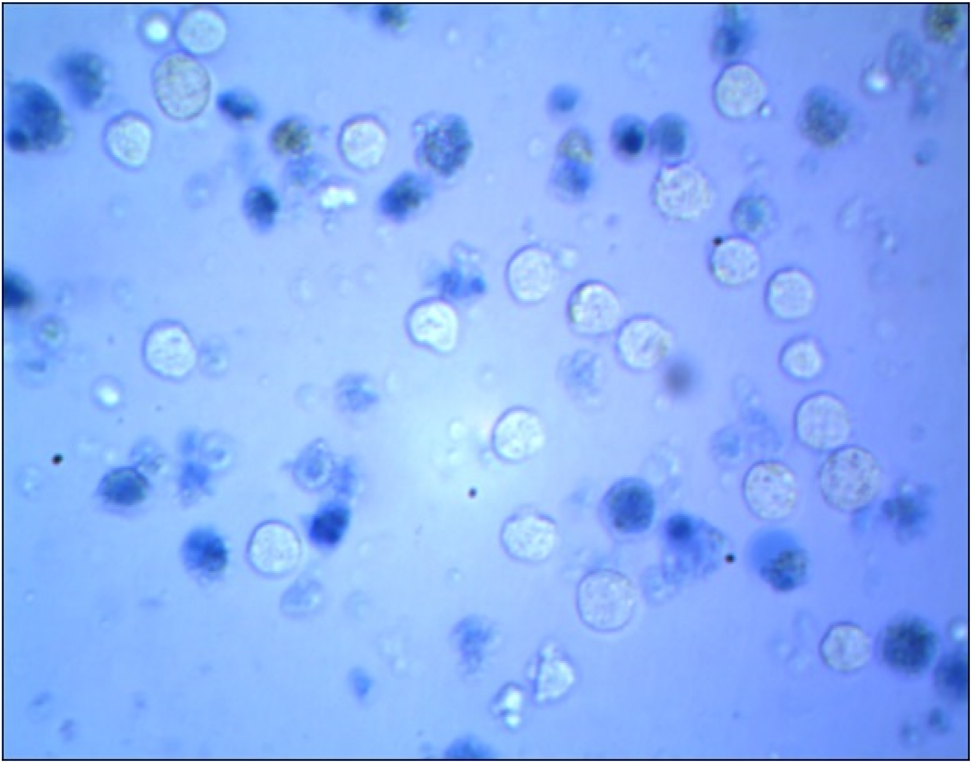
(b) Microscopic image of Cu(ii)(HPAN)_2_-treated MOLT-4 cells incubated for 48 hours at concentration 20 μM.

The affiliated institution (Gurudas College, Kolkata, India) has reviewed the original raw data and confirmed its reliability and integrity.

This notice supersedes the information provided in the expression of concern related to this article.

The Royal Society of Chemistry apologises for these errors and any consequent inconvenience to authors and readers.

